# Riding in silence: a little snowboarding, a lot of small RNAs

**DOI:** 10.1186/1758-907X-1-8

**Published:** 2010-03-15

**Authors:** Stefan L Ameres, Ryuya Fukunaga

**Affiliations:** 1 Department of Biochemistry and Molecular Pharmacology, Massachusetts Medical School, 364 Plantation Street, Worcester, MA 01605, USA

## Abstract

The recent symposium, *RNA silencing: Mechanism, Biology and Applications*, organized by Phillip D. Zamore (University of Massachusetts Medical School) and Beverly Davidson (University of Iowa), and held in Keystone, Colorado, brought together scientists working on diverse aspects of RNA silencing, a field that comprises a multitude of gene regulatory pathways guided by microRNAs, small interfering RNAs and PIWI-interacting RNAs.

## Review

From 14 to 19 January 2010, small RNAs once again attracted the attention of more than 500 attendees to symposium on *RNA silencing: Mechanism, Biology and Applications*, held in Keystone, Colorado. In the midst of the breathtaking panorama of the Rocky Mountain summits, exciting and rapidly evolving science mixed with superb riding and skiing created a stimulating atmosphere for discussions on the mechanisms, biology and applications of RNA silencing.

### Biogenesis of small RNAs

A much-discussed topic at the meeting was the mechanisms and regulation of micro (mi)RNA biogenesis. In her keynote address, Narry Kim (Seoul National University) elaborated on the effect of pre-miRNA uridylation. She reported that the terminal uridylyl transferase, TUT4, acts on the 3' termini of pre-miRNAs to suppress pre-miRNA processing [1,2]. Although this process requires the scaffold protein Lin-28 to recruit the TUTase to specific pre-miRNAs, uridylation is not limited to these pre-miRNAs. Whereas oligo-uridylylation inhibits pre-miRNA dicing, mono-uridylylation can enhance pre-miRNA processing by human and fly Dicer proteins *in vitro*, suggesting that post-transcriptional pre-miRNA modification can have a versatile effect on processing. Surprisingly, the addition of one or a few additional nucleotides to the 3' end of a pre-miRNA did not alter the 3' end of the miRNA excised from the 5' side of the stem. To explain these data, Kim proposed an alternative model to the PAZ-anchored molecular ruler model that emerged from the structure of *Giardia *Dicer [3]. She suggested that Dicer proteins in higher eukaryotes measure mainly from the 5' end of a pre-miRNA, rather than from its 3' end.

Jennifer Doudna (University of California, Berkeley) described the first structural insights into human pre-miRNA processing and loading by electron microscopy [4]. She presented biochemical cross-linking studies employing a recombinant human Dicer-TRBP (trans activation response RNA binding protein) complex. These data suggest that the human proteins, 'sense' the thermodynamic asymmetry of the ends of a small RNA duplex, as was originally proposed for their fly counterparts, *Drosophila *Dicer-2 and R2D2 [5]. For both the human and fly proteins, the double-stranded (ds)RNA binding partner of Dicer binds to the more stable end of a small interfering (si)RNA duplex, directing the preferential loading into Argonaute of the siRNA strand, whose 5' end is less stably paired. PACT (protein activator), the other dsRNA-binding partner of human Dicer, showed similar behavior in these studies. Despite this similarity, the two dsRNA-binding partners of Dicer had opposite effects on pre-miRNA processing: TRBP activated dicing thus increasing turnover, whereas PACT inhibited dicing. Thus dsRNA-binding dicer partner proteins may tune Dicer activity.

Dinshaw Patel (Memorial Sloan-Kettering Cancer Center) presented the structure of a budding yeast Dicer protein from *Saccharomyces castellii*. This 'Dicer' lacks a PAZ domain, which normally allows Dicer to recognize the two-nucleotide 3' overhang of small RNA precursors, and contains only a single RNase III domain. The structure revealed that the protein dimerizes to generate two catalytically active centers, with each having the potential to cleave one strand of the dsRNA, leaving duplex ends with overhangs two nucleotides in length. The uniform product length (23 nucleotides) observed *in vitro*, may reflect dimer-dimer interactions on duplex RNA, a novel strategy to assure a uniform length of Dicer products in a small RNA pathway.

A hitherto underappreciated feature of Dicer proteins in higher eukaryotes is the helicase domain, the function of which is not understood. Brenda Bass (University of Utah) showed that in *Caenorhabditis elegans *the helicase domain is dispensable for pre-miRNA processing; expression of a helicase mutant Dicer in Dicer null mutant animals rescued the phenotype caused by loss of miRNAs. However, deep sequencing revealed that Dicer helicase mutants lose primary endo-siRNAs. *In vitro *processing experiments suggest that the structure of endo-siRNA precursor dsRNAs versus the structure of pre-miRNAs might be the discriminating factor, because dsRNA bearing a blunt end or a 5' overhang could not be processed by a Dicer lacking a functional helicase domain.

The initial step in miRNA biogenesis, primary (pri)-miRNA processing, can be modulated by accessory proteins such as SMAD proteins, as reported by Brandi Davis (Tufts University) [6]. Davis described experiments that suggest that SMAD proteins associate with p68, Drosha and DGCR8 on the pri-miRNA to promote conversion of the pri-miRNA into a pre-miRNA. The recognition step might rely on a conserved sequence in the center of the designated mature miRNA.

In the second keynote address, James Carrington (Oregon State University) talked about the mechanisms that produce *trans*-acting siRNA (tasi-RNA) in plants [7-9]. The association of miR-173 with Ago1 and miR-390 with Ago7 is required to generate precise ends on TAS1 or TAS3 loci transcripts, respectively, and are required for the recruitment of an RNA-dependent RNA polymerase to generate dsRNA that is then further processed into tasi-RNAs. Besides the importance of the 5' nucleotide of a miRNA in determining its association with particular Argonaute proteins, the length of miRNAs also seems to be important for tasi-RNA formation: miRNAs involved in generation of tasi-RNAs are predominantly 22 nucleotides long, whereas plant miRNAs in general are mostly 21 nucleotides long. Interestingly, shortening miR-173 from 22 to 21 nucleotides interfered with the generation of tasi-RNAs from the TAS1 locus, although neither its loading into Ago1 nor its ability to cleave the TAS1 transcript was altered.

### miRNA function

A series of talks described new insights into the mechanism of small RNA function in various organisms. David Bartel (Massachusetts Institute of Technology) presented compelling genome-wide support for the recent idea that much of the repression mediated by miRNAs reflects mRNA destabilization rather than translational repression [10,11]. He described ribosome-profiling experiments [12] in the presence or absence of specific miRNAs. Changes in ribosome-protected fragments mostly reflected changes in mRNA levels, whereas there was only a small, although detectable, change in ribosome density on miRNA-regulated transcripts. This was much of a surprise to most in the audience, because translational repression has long been considered to be the major mechanism of miRNA function. A lively and extended question and answer session followed the talk!

Phillip Zamore (University of Massachusetts Medical School) speculated on the puzzling discrepancy between the nature of miRNA targets in plants and animals. In plants, miRNAs usually pair with high or perfect complementarity to their targets, whereas in animals pairing is usually limited and is often restricted to only the 'seed region' (nucleotides 2 to 8) of the miRNA guide. He showed that in *Drosophila*, extensive complementarity between miRNAs and their target RNAs leads to tailing and degradation of miRNAs. Interestingly, flies have a small RNA protection system that is based on the methyltransferase Hen1, and methylates small RNAs associated with Ago2, the effector protein in the RNAi pathway. The methylation of small RNAs at their 3' terminal ribose counteracts tailing and degradation, and is required to ensure the integrity and abundance of endogenous small RNAs in *Drosophila*. In plants, all miRNAs are methylated, which might have allowed high-complementarity targets to evolve. Interestingly, the target-dependent small RNA tailing and degradation machinery seems to be conserved in humans.

Tudor Fulga (Harvard Medical School) introduced a powerful tool to study miRNA function in flies [13]. The expression of miRNA sponges (originally established by Phil Sharp's group in mammalian cell lines [14]) allows uncovering of miRNA null mutant phenotypes with spatiotemporal resolution in transgenic flies employing the Gal4-UAS system. Although it is still unclear if miRNA sponges work via competition or miRNA degradation, this system will be a valuable tool to understand miRNA function in model organisms such as *Drosophila melanogaster*.

Victor Ambros (University of Massachusetts Medical School) gave an update on how the tripartite motif (TRIM)-NHL protein, NHL-2, functions as a cofactor for miRNA-induced silencing by enhancing post-transcriptional repression of target mRNAs [15]. NHL-2 associates with CGH-1 (a P-body component) and the Argonaute proteins, ALG-1 and ALG-2, and these interactions are ATP-dependent. Dr Ambros also reported novel *alg-1 *mutants identified in a screen for genetic suppressors of *lin-28*. These *alg-1 *mutations affect amino acids in or surrounding the PIWI domain and act as antimorphic mutations because they have a more detrimental effect on the organism than does a null mutant. Such an effect might be explained by a possible competition of mutant Alg-1 with Alg-2, which in an *alg-1 *null mutant can take over miRNA regulation.

Martijn Kedde (Netherlands Cancer Institute) described a switch in RNA structure in the 3' untranslated region (UTR) of p27 that regulates miRNA access to its binding site. He suggested that the RNA-binding protein, Pumilio, binds to the p27 3' UTR in cycling cells, opening a hairpin structure and thereby increasing the accessibility of miRNA binding sites to miR-221 and miR-222. Growth factor stimulation changes the phosphorylation status of Pumilio, which modifies its affinity for the p27 3' UTR binding sites. This might contribute to the upregulation of p27 in quiescent cells, providing the basis for a novel RNA-binding protein-induced switch that modulates miRNA-mediated gene regulation.

In the first part of his talk, Dinshaw Patel presented structural insights into the ternary interaction and structural rearrangements associated with the *Thermus thermophilus *Argonaute protein, its associated guide DNA, and a target RNA [16]. He also gave an outlook on a novel structure of the bacterial Argonaute protein bound to an uncleaved DNA target, which allowed investigation of rearrangements at the catalytic center of the wild-type protein. He found that upon formation of 15 perfect base pairs between the DNA guide and its target, Argonaute undergoes conformational transitions that lock it into a catalytically competent state, positioning a divalent cation near the scissile phosphate.

Eliza Izaurralde (Max Planck Institute for Developmental Biology) reported that the interaction between PABC1 and the silencing domain of GW182 results in competition for eIF4G binding to the 5' cap, ultimately interfering with mRNA circularization [17]. This increases the accessibility of the mRNA 3' end to deadenylating enzymes, contributing to miRNA-mediated destabilization of mRNAs.

Gene Yeo (University of California, San Diego) discussed the recent identification of endogenous Argonaute binding sites in *C. elegans *using cross linking, ALG1 immunoprecipitation, and high-throughput sequencing (HITS) (with crosslinking immunoprecipitation (HITS-CLIP or CLIP-Seq)) [18]. Yeo and his collaborators are currently trying to identify Argonaute binding sites in human stem cells, which exhibit miRNA expression profiles distinct from common human cell lines.

### Biology of small RNAs

Several talks at the meeting reiterated the multifarious character of miRNAs, being involved in the regulation of a plethora of biological processes. In the second part of her keynote lecture, Narry Kim reported that miR-8/miR-200 regulate body weight homeostasis in flies and mice [19]. miR-8 in flies and miR-200 in humans target USH (Usher syndrome)/FOG2 (friend of Gata 2), which in turn inhibits phosphoinositide 3-kinase (PI3K). Loss of those miRNAs therefore activates fat cell growth through increased PI3K activity. Their study identified two novel regulators of insulin signaling, USH/FOG2 and miR-8/miR-200.

Craig Mello (University of Massachusetts Medical School) presented an historic overview of small RNA pathways in *C. elegans*, emphasizing a class of endo-siRNAs called 22G-RNAs. Several distinct 22G-RNA systems act in worms. In the germline, one system requires worm-specific AGOs, including WAGO-1, an Argonaute that localizes to germ line nuage structures and silences certain genes, transposons, pseudogenes and cryptic loci [20]. In association with the Argonaute CSR-1, 22G-RNAs localize to chromosomes and are required for proper chromosome segregation [21].

Phil Sharp (Massachusetts Institute of Technology) talked about the role of miRNAs in mammalian cell robustness. He showed that although misexpression of miRNAs was repeatedly found to be associated with certain types of cancer, miRNA regulation is not essential for cancer. A tumor cell line that is defective for miRNA synthesis can still form tumors. In a reporter cell line, Dr Sharp tested the threshold for miRNA-mediated repression under increased transcription rates and found that at low levels of reporter expression, miRNA-mediated repression is strongest, and that high expression almost completely relieves this type of regulation. Finally, he showed that during stress, the cytoplasmic subcellular localization of mRNAs and miRNAs is dependent upon formation of poly(ADP-ribose) and that miRNA repression is reduced under these conditions.

Deepak Srivastava (Gladstone Institute of Cardiovascular Disease) talked about miRNA regulation of cardiovascular cell fate. He elaborated on individual miRNAs that govern differentiation and development of embryonic stem cell-derived and induced pluripotent stem cell-derived multipotent cardiac progenitors into cardiomyocytes, endothelial and smooth muscle cells, thereby emphasizing the potential of miRNA manipulation in influencing the outcome of cell fates and of cellular homeostasis.

Mark Boldin (Regulus Therapeutics) showed that miR-146a is an endotoxin-responsive miRNA, and miR-146 knockout mice are hypersensitive to bacterial challenge and develop a spontaneous autoimmune-like disorder. He suggested that miR-146a serves as a molecular brake for both the inflammatory response and oncogenic transformation of immune cells.

Dasaradhi Palakodeti (University of Connecticut Health Center) discussed the involvement of small RNAs in the regenerative capabilities of the planarian *Schmittea mediterrenea*. The regeneration of lost tissues and organs from neoblasts (the equivalents of stem cells in higher organisms) is sensitive to irradiation, which depletes the animals of neoblasts. Knockdown of *Smed*-Ago1 by RNA interference (RNAi) leads to drastic regeneration defects and a decrease in the neoblast population, implicating the miRNA pathway in regeneration and neoblast function in planaria.

### Small RNAs in chromatin formation

Sarah Elgin (Washington University) discussed the diminutive *Drosophila melanogaster *'dot' chromosome, which is mainly occupied by heterochromatin bearing HP1 and H3K9 dimethylation, causing variegated expression of transgenes inserted into diverse regions of the fourth chromosome. Silencing of reporters inserted into the 4th chromosome can be suppressed by mutations in *aubergine *and *piwi*. HP1 binds to PIWI *in vitro*, and in the germline, PIWI is needed for HP1 localization at the promoter region of *HeT-A *transposons.

Shiv Grewal (National Institutes of Health) showed that in *Schizosaccharomyces pombe*, the histone methyltransferase Clr4 is required for generation of centromeric siRNAs, partly independent of its role in methylation of histone H3 lysine 9. He presented evidence suggesting that the Clr4 complex and the RNAi effector complex, RNA-induced transcriptional silencing (RITS), might be recruited to chromatin in a cooperative manner. He also showed that loss of either Clr4 or Ago1 in the absence of functional H2A.Z increases the levels of antisense transcripts, which are usually suppressed with assistance of Dicer and the exosome. He proposed a model in which heterochromatin and RNAi components cooperate with a variant histone H2A.Z to suppress non-coding and antisense transcripts.

Danesh Moazed (Harvard Medical School) described Dicer-independent 'primal small RNAs' (priRNAs) in *S. pombe *[22]. These 22-23-nucleotide RNAs posses a 5' uracil bias and specifically associate with Argonaute1. priRNAs map to the 3' UTR of mRNAs and to non-coding RNAs in centromeric repeat regions, where they mediate low levels of H3K9 methylation. Convergent transcripts from centromeric repeats might give rise to antisense priRNAs, which cause siRNA amplification through the RNA-dependent RNA polymerase complex and Dicer-1.

Stephan Emmerth (Friedrich Miescher Institute) showed that Dicer-1 in the fission yeast *S. pombe *is localized to the nucleus and is enriched in the nuclear periphery [23]. Localization requires a short motif, which constrains nuclear export promoted by the dsRNA binding domain of Dicer-1. Deletion of this motif results in localization of Dcr1 to the cytoplasm and causes marked changes in gene expression and failure to assemble heterochromatin.

### RNAi and therapeutics

Beverly Davidson (University of Iowa) talked about therapeutic strategies to target Huntington's disease (HD). Preliminary *in vivo *experiments in established mouse models of this fatal neurodegenerative disorder indicated disease improvement after silencing both mutant and wild-type *huntingtin *alleles via RNAi. This could provide an important alternative approach to the as yet untested single nucleotide polymorphism-based therapy, which may be useful in ~75% of patients with HD. In additional work, she showed that dysregulation of miRNAs in brains from patients with HD might contribute to its varying clinical presentations and disease course.

Priti Kumar (Yale School of Medicine) presented progress in strategies for delivering siRNAs into T cells for antiviral therapy. She described a CD7-specific single-chain antibody conjugated to oligo-9-arginine peptide [24] or lymphocyte function-associated antigen (LFA)-1 integrin antibody conjugated to stabilized nanoparticles [25].

Daniel G. Anderson (Massachusetts Institute of Technology) elaborated on the combinatorial development of nucleic acid delivery agents using a high throughput approach to develop biomaterials. He provided updates on the second generation of siRNA delivery formulations, which allow efficient knockdown *in vivo *by doses as low as 0.03 mg/kg in non-human primates [26]. He also showed preliminary performance tests for simultaneous knockdowns of up to 10 genes.

Sakari Kauppinen (Santaris Pharma) described tiny locked nucleic acid (LNA) antisense oligonucleotides targeting the seed of miRNAs to repress their function. An LNA-anti-miR (SPC3649) targeting miR-122 in the liver has previously been used in mice and non-human primate studies to induce a long-lasting decrease in serum cholesterol, and the success story of this LNA compound continues, because the same strategy has been introduced as a new anti-hepatitis C virus therapy in chronically infected chimpanzees [27]. Clinical trials for SPC3649 are ongoing, and Kauppinen reported that the molecule seems to be well tolerated and shows efficacy in healthy human volunteers.

### PIWI-interacting (pi)RNAs

Mikiko Siomi (Keio University School of Medicine) described the specific association of a Tudor domain-containing protein, Tudor, with Aubergine and Ago3 through symmetric dimethyl-arginine (sDMA) modifications in the PIWI proteins introduced by dPRMT5/Capsuleen/DART5. She suggested that this might be involved in the loading of primary PIWI-interacting (pi)RNAs into the effector PIWI proteins [28]. Studies in a fly ovarian somatic cell line expressing PIWI but not Aubergine or Ago3, indicate that PIWI and the RNA helicase Armitage (Armi) but not Maelstrom (Mael) or Spindle-E (Spn-E) (both factors implicated in the piRNA pathway in flies) are required for piRNA accumulation. Armi co-localizes with the helicase and Tudor domain-containing protein, Yb, to distinct foci in the cytoplasm called Yb bodies. Similar colocalization can be observed in fly ovaries and testes. In a model, PIWI and Armi were proposed to be involved in primary piRNA accumulation in Yb bodies, whereas Mael and Spn-E might participate in the 'ping-pong' amplification loop.

René Ketting (Royal Netherlands Academy of Arts and Sciences) presented novel insights into the zebrafish piRNA pathway. Ziwi and Zili, the two zebrafish PIWI proteins, localize to nuage, a germline-specific perinuclear structure, and associate with antisense and sense piRNAs, respectively, exhibiting the characteristic ping-pong signature. As in other animals, zebrafish piRNAs bear a 2'-*O*-methyl group on their 3' terminal nucleotide, which protects them from uridylation and destabilization. Interestingly, mutants in the methyltransferase, Hen1, only develop into males, which are fertile. This is in contrast to Piwi mutant males, which are sterile, suggesting that in *hen1 *mutants the piRNA pathway is disturbed rather than disrupted. Again as in flies, the two zebrafish Piwi proteins bear sDMA modifications required for binding to Tdrd1. Tdrd1 associates with piRNA precursor transcripts and thus may function in substrate selection. Furthermore, PIWI proteins are required for meiosis and cell differentiation in a manner that is not related to transposon silencing.

Alex Bortvin (Carnegie Institution of Washington) suggested that an evolutionarily conserved gene, *maelstrom*, is needed for transposon silencing in the male mouse germline, and for proper chromosome synapsis and crossing over in both male and female meiosis [29].

## Conclusions

The conference highlighted the expanding roles of small-silencing RNAs in various biological systems, their finely tuned biogenesis, regulatory mechanisms and potential as therapeutics. It is exciting to see that after a decade of intensive study, the field is not running out of surprises. In fact, many of the presentations raised captivating questions that left the attendees of the meeting yearning for next year's conference.

## Competing interests

The authors declare that they have no competing interests.

## Authors' contributions

SLA and RF wrote, read and approved the final manuscript.
